# Necessity of Microdissecting Different Tumor Components in Pulmonary Tumor Pyrosequencing

**DOI:** 10.1155/2016/8759267

**Published:** 2016-08-11

**Authors:** Dahui Qin, Zhong Zheng, Shanxiang Shen, Prudence Smith, Farah K. Khalil

**Affiliations:** Department of Pathology, Moffitt Cancer Center, 12902 USF Magnolia Drive, Tampa, FL 33612, USA

## Abstract

Microdissection is a useful method in tissue sampling prior to molecular testing. Tumor heterogeneity imposes new challenges for tissue sampling. Different microdissecting methods have been employed in face of such challenge. We improved our microdissection method by separately microdissecting the morphologically different tumor components. This improvement helped the pyrosequencing data analysis of two specimens. One specimen consisted of both adenocarcinoma and neuroendocrine components. When both tumor components were sequenced together for KRAS (Kirsten rat sarcoma viral oncogene homolog) gene mutations, the resulting pyrogram indicated that it was not a wild type, suggesting that it contained KRAS mutation. However, the pyrogram did not match any KRAS mutations and a conclusion could not be reached. After microdissecting and testing the adenocarcinoma and neuroendocrine components separately, it was found that the adenocarcinoma was positive for KRAS G12C mutation and the neuroendocrine component was positive for KRAS G12D mutation. The second specimen consisted of two morphologically different tumor nodules. When microdissected and sequenced separately, one nodule was positive for BRAF (v-raf murine sarcoma viral oncogene homolog B1) V600E and the other nodule was wild type at the BRAF codon 600. These examples demonstrate that it is necessary to microdissect morphologically different tumor components for pyrosequencing.

## 1. Introduction

Microdissection has been used to obtain tumor tissue for molecular testing with the primary goal of separating tumor and normal tissue to increase the amount of tumor DNA and, therefore, increasing test sensitivity. Tumor heterogeneity imposes new challenges for tissue sampling. We need to microdissect tumor not only from normal tissue but also from different intratumor elements. Different methods have been used for microdissection [1, 2].

Pyrosequencing is a sequencing technology that is more sensitive than Sanger sequencing [3, 4]. Pyrosequencing results are usually depicted as a series of peaks called a pyrogram, which reflects the DNA sequence. Pyrosequencing has been used in detecting different mutations, including KRAS (Kirsten rat sarcoma viral oncogene homolog) [5]. In our lab, it is used to screen for mutations in KRAS, EGFR (epidermal growth factor receptor), and BRAF (v-raf murine sarcoma viral oncogene homolog B1) since these gene mutations are associated with gene targeted therapies [6, 7]. For example, EGFR exon 19 deletions are associated with the tumor susceptibility to EGFR targeted therapy while KRAS mutations are associated with the tumor resistance to EGFR targeted therapy. BRAF V600E mutation is associated with the tumor susceptibility BRAF targeted therapy [6, 7]. In this study, we report unusual molecular test results from two different specimens (specimens 1 and 2), which demonstrate the necessity of microdissecting morphologically different components prior to pyrosequencing.

## 2. Material and Methods

Two separate specimens were examined for this study with distinctly different morphologies:

1. Adenocarcinoma with neuroendocrine differentiation (specimen 1).
2. Adenocarcinoma with two morphologically different nodules (specimen 2).

### 2.1. Microdissection of the Adenocarcinoma with Neuroendocrine Differentiation

Specimen 1 consists of a 3-cm pulmonary nodule, containing adenocarcinoma and neuroendocrine components. The formalin fixed paraffin embedded (FFPE) tumor tissue sections were microdissected manually using two methods: one combining both adenocarcinoma and neuroendocrine components and the second separating the adenocarcinoma and neuroendocrine components. The three microdissected samples from specimen 1 were sequenced separately.

### 2.2. Microdissection of the Adenocarcinoma with Morphologically Different Nodules

Specimen 2 consists of adenocarcinoma with two morphologically different pulmonary nodules. One tumor nodule is invasive adenocarcinoma, solid predominant type [8], and the other is minimally invasive adenocarcinoma, nonmucinous tumor [8]. Each nodule was microdissected separately and sequenced separately.

### 2.3. DNA Extraction

DNA was extracted from the microdissected tissue, using the QIAamp DNA FFPE Tissue Kit (Qiagen, Cat# 56404, Valencia, CA 91355, USA) and QiaCube instrument (Qiagen, Valencia, CA 91355, USA). The microdissected tissue was transferred into a 2 mL tube containing 180 *μ*L of ATL buffer. Next, twenty microliters of proteinase K was then added to the tube and mixed by vortexing. The tissue mixture was incubated at 56°C for 2 hours followed by 90°C incubation for 1 hour. Following incubation the sample was loaded onto the QiaCube to complete the rest of DNA extraction process using the gDNA Extraction Program for FFPE tissue. Finally, the DNA extract was eluted into 100 *μ*L ATE buffer to be used for pyrosequencing.

### 2.4. Pyrosequencing

Pyrosequencing for KRAS, EGFR, and BRAF mutation was performed according to the manufacturer's instructions [9–11] with some modifications. The targeted DNA sequences that included KRAS codons 12, 13, and 61, EGFR exons 18, 19, 20, and 21, and BRAF codon 600 were amplified using Qiagen KRAS, EGFR, and BRAF mutation test kits. The PCR products were used as templates for sequencing. Sequencing primers from the Qiagen KRAS, EGFR, and BRAF kits are designed to hybridize to the sequence near the targeted mutations, usually within a few nucleotides.

**Figure 1:**
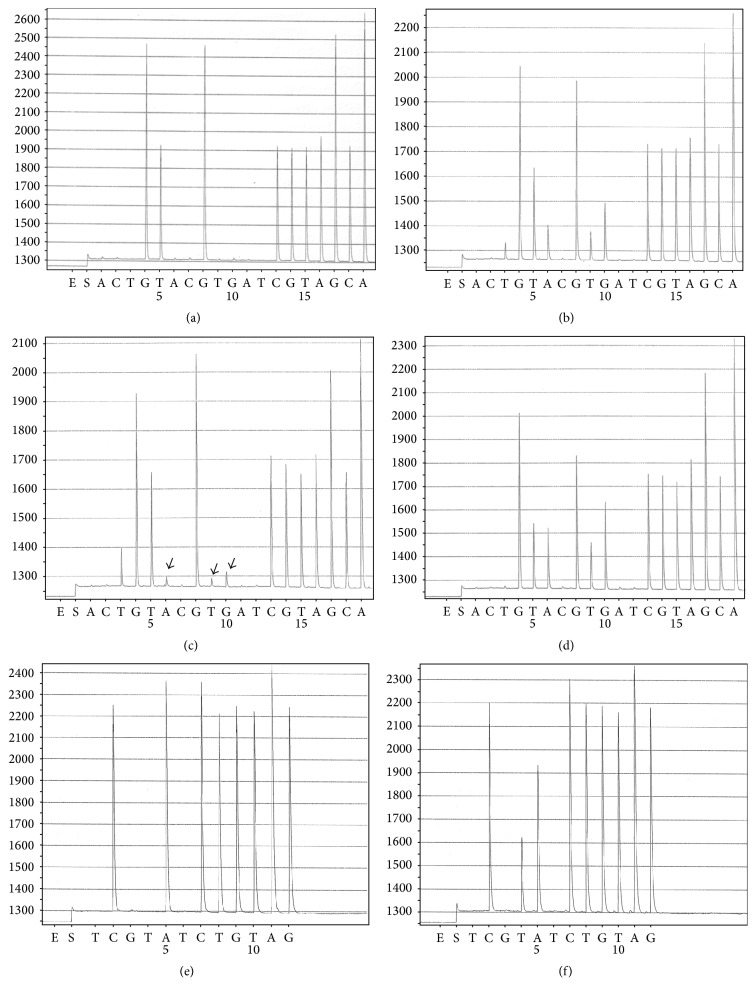
Six pyrograms, panels (a, b, c, d, e, and f), are present. The *x*-axis represents pyrosequencing dispensing order. The *y*-axis represents peak height. Panel (a) shows a pyrogram of wild type KRAS codons 12 and 13. Panel (b) shows a pyrogram from the whole tumor of an adenocarcinoma with neuroendocrine differentiation. The pyrogram peak pattern is different from a wild type pattern, but the pattern cannot match any KRAS mutation patterns. Panel (c) shows a pyrogram from the adenocarcinoma component that is shown in Figure 2(a), indicating KRAS G12C mutation. Three small peaks (indicated by black arrows) reflect the contamination of adenocarcinoma by neuroendocrine component due to imperfect microdissection. Panel (d) shows a pyrogram from the neuroendocrine component that is shown in Figure 2(b), indicating KRAS G12D mutation. Panel (e) shows a pyrogram from a tumor nodule with features of minimally invasive adenocarcinoma that is shown in Figure 2(d), indicating a wild type BRAF. Panel (f) shows a pyrogram from an invasive adenocarcinoma, solid predominant, poorly differentiated carcinoma nodule, which is shown in Figure 2(c), indicating BRAF V600E mutation.

The targeted sequence for KRAS codons 12 and 13 is GGTGGCGTAGG and the dispensing order is ACTGTACGTGATCGTAGCA (Figure 1(a), *x*-axis). The targeted sequence for KRAS codon 61 is ACAGCAGGTCAAGAG. Since the sequencing primer for KRAS codon 61 is a reverse primer, the pyrosequencing reading is the reverse complement of the target sequence, CTCTTGACCTGCTGT. Therefore the dispensing order is GCTCGATACGACCT (data not shown). A typical pyrogram peak pattern for wild type KRAS codons 12 and 13 is shown in Figure 1(a).

The targeted sequence for BRAF codon 600 is CTAGCTACAGTG. The sequence primer for BRAF codon 600 is also a reverse primer; therefore the pyrosequencing reading for BRAF is the reverse complement of the targeted sequence, CACTGTAGCTAG. The dispensing order is TCGTATCTGTAG (Figure 1(e), *x*-axis). A typical pyrogram peak pattern for wild type BRAF codon 600 is shown in Figure 1(e).

EGFR exons 18, 19, 20, and 21 were also sequenced (data not shown). The targeted sequences were GGCTCCGGTGC (exon 18), TATCAAGGAATTAAGAGAAGCAACATCTCCGAAAG (exon 19), CAGCGTGG and ATCACGCAG (exon 20, codons 768 and 790), and CTGGCCAAACTGCTGGGT (exon 21) respectively. The dispensing orders for EGFR exons are CATGTCACTCGTG (for exon 18), CTATCACTGTCAGCTCGATCGTCATCGTCACGC (for exon 19), GCAGTACGTGTCGTGTACGTGACCACACTG and GATCATCTG (for exon 20, codons 768 and 790 resp.), and ACGTGTCACATGTC (for exon 21).

## 3. Result

### 3.1. Morphology and KRAS Mutations of the Adenocarcinoma with Neuroendocrine Differentiation (Specimen 1)

**Figure 2:**
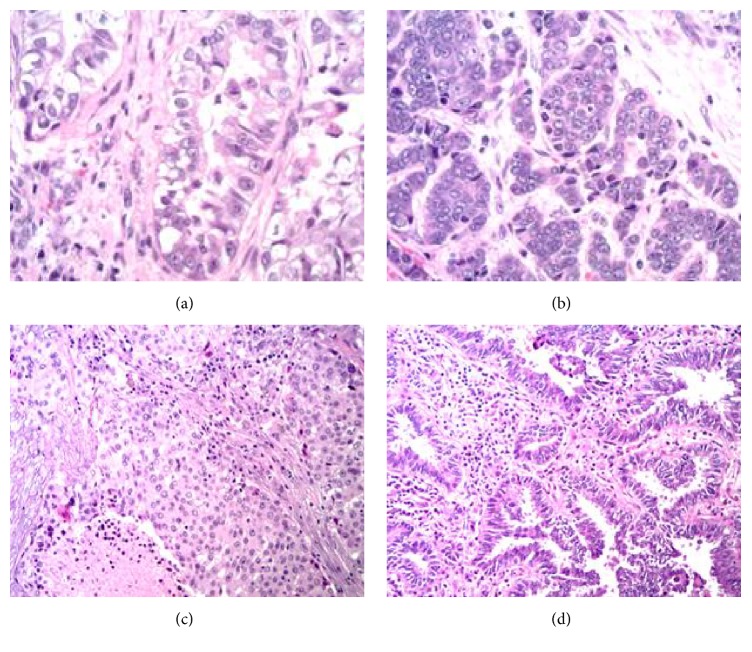
H&E stain of two specimens is shown. Panel (a) shows the glandular tumor component from specimen 1. Panel (b) shows the neuroendocrine tumor component from specimen 1. Panel (c) shows an invasive adenocarcinoma, solid predominant, poorly differentiated tumor, from specimen 2. Panel (d) shows the minimally invasive adenocarcinoma, nonmucinous type, from specimen 2.

The tumor morphology of specimen 1 is shown in Figures 2(a) and 2(b). The tumor consists of both glandular component (Figure 2(a)) and solid nested neuroendocrine component (Figure 2(b)). Immunohistochemical stains, performed with appropriate controls, revealed positivity for neuroendocrine marker, chromogranin, in the solid nests, and no reactivity for the marker in the glandular component (data not shown).

The pyrosequencing result of the entire tumor from specimen 1, including both adenocarcinoma and neuroendocrine components, is shown in Figure 1(b). The pyrogram of this result is different from that of wild type (shown in Figure 1(a)), suggesting that this pattern may reflect a mutated KRAS. The pattern, however, does not match the peak pattern of any KRAS mutations. Sequencing was then performed on each of the microdissected adenocarcinoma and neuroendocrine components. The pyrograms now show that the adenocarcinoma is positive for KRAS G12C mutation (Figure 1(c)) and the neuroendocrine component is positive for KRAS G12D mutation (Figure 1(d)). Both tumor components were negative for BRAF and EGFR mutations (data not shown).

### 3.2. Morphology and BRAF Mutation of the Adenocarcinoma with Morphologically Different Nodules

Specimen 2 consists of two tumor nodules, invasive adenocarcinoma, solid predominant type [8] (Figure 2(c)), and minimally invasive adenocarcinoma, nonmucinous tumor [8] (Figure 2(d)). Immunohistochemical stains performed with appropriate controls on both tumor nodules reveal immunoreactivity for TTF-1 (Thyroid Transcription Factor-1, data not shown), indicating pulmonary primary. Pyrosequencing result of the first nodule is positive for BRAF V600E mutation (Figure 1(f)), and the second nodule is negative for BRAF codon 600 mutation (Figure 1(e)). Both tumor nodules are negative for KRAS and EGFR mutations (data not shown).

## 4. Discussion

Tumor heterogeneity has been previously recognized and vigorously studied. The heterogeneity involves different levels of tumor clonal evolution, including cellular morphology, gene mutations, and biological responses to therapies [12, 13]. Tumor heterogeneity imposes a challenge to tissue sampling in molecular testing. In our practice, it has been noted that a tumor specimen may have morphologically different components, like specimen 1 in this report or morphologically different tumor nodules, as in specimen 2.

Specimen 1 consists of adenocarcinoma and neuroendocrine components. Morphologically, the two components are intermingled in some areas of the tumor but are distinct in other areas of the tumor (Figures 2(a) and 2(b)). When the entire tumor was microdissected altogether for mutation analysis, the data was difficult to interpret (Figure 1(b)). The pyrogram pattern was different from wild type pattern (Figure 1(a)), but it did not match any KRAS mutation pattern. In fact, the pyrogram pattern could not be interpreted. When the adenocarcinoma and neuroendocrine components were microdissected separately and tested separately for KRAS mutations, the adenocarcinoma component was found to harbor a KRAS G12C mutation (Figure 1(c)), and the neuroendocrine component was found to harbor a KRAS G12D mutation (Figure 1(d)). Retrospectively, the pyrogram from the whole tumor (Figure 1(b)) actually reflects the overlap of KRAS G12C and G12D pyrograms. This explains why the pyrogram from the whole tumor could not be interpreted as any specific KRAS mutation. Only when the two tumor components were tested separately did it become clear that each component harbored a different KRAS mutation.

In Figure 1(c), three small peaks are present (indicated by arrows). These small peaks are due to imperfect microdissection and reflect contamination of the adenocarcinoma by the neuroendocrine component. The results of these molecular tests indicate that it is necessary to microdissect morphologically different components separately prior to pyrosequencing to ensure accurate interpretation.

Specimen 2 consists of two morphologically different tumor nodules, an invasive adenocarcinoma, solid predominant type [8] (Figure 2(c)), which is positive for BRAF V600E mutation (Figure 1(f)) and a minimally invasive adenocarcinoma, nonmucinous tumor (Figure 2(d)), which is negative for BRAF codon 600 mutation (Figure 1(e)). If only the minimally invasive adenocarcinoma had been tested, we would have mistakenly assumed that the patient's tumor had no BRAF mutation. Likewise, if only the invasive adenocarcinoma, solid predominant nodule, had been tested, we would have believed that both tumor nodules contained the identified BRAF mutation. In either case, the molecular test report would not accurately reflect whole picture of the BRAF gene mutation status, again emphasizing the necessity to microdissect each tumor nodule/component separately for mutation testing.

In current pathology practice, both small specimen size and tumor heterogeneity complicate the sampling process, indicating an increasing need for different microdissecting methods. A microdissection of different tumor components, as discussed in this report, addresses a portion of the difficulties in tissue sampling. This approach works when the heterogeneous components are morphologically different. Different methods have been and are still being developed to address different aspects of the tissue sampling issue. For example, microdissecting heterogeneous tumor components based on immunohistochemical phenotypes has been recently reported [2].

Different methods of microdissecting have improved the quality of clinical molecular testing and have a direct impact on patient care as indicated by the results from specimen 1. This specimen was an invasive adenocarcinoma with neuroendocrine differentiation. At the beginning, the entire tumor was microdissected altogether and the result was not interpretable. The puzzle was not resolved until the two tumor components were microdissected and tested separately. Then it became clear that two components bear different KRAS mutations.

Specimen 2 is an example that microdissecting different tumor components may have impact on patient management. This case presents two tumor nodules of different morphology. One was an invasive adenocarcinoma, solid predominant type, and the second nodule was a minimally invasive adenocarcinoma. The former was positive for BRAF V600E mutation and the latter was wild type for BRAF. Treatment for tumors with or without BRAF mutation could be different. Microdissecting and testing each tumor component separately can provide more precise mutation information for the patient's personalized management.
